# Supplementary material for: Acidic Urine pH and Clinical Outcome of Lower Urinary Tract Infection in Kidney Transplant Recipients Treated with Ciprofloxacin and Fosfomycin
Source: Antibiotics (Basel). 2024 Jan 24;13(2):116. doi: 10.3390/antibiotics13020116 (PMC10886300; doi:10.3390/antibiotics13020116)
Supplement: Supplementary file 1 [file antibiotics-13-00116-s001.zip › antibiotics-2839367-supplementary.pdf]

Title: Acidic urine pH and clinical outcome of lower urinary tract infection in kidney transplant recipients treated with ciprofloxacin and fosfomycin

Supplementary data

Supplementary Tables S1 to S13

Supplementary Table S1. Demographics and characteristics of the 115 kidney transplant recipients (KTR) with urinary tract infection (UTI) by *Escherichia coli*, and according the asymptomatic bacteriuria (AB) and cystitis episodes.

Variables	*E. coli* 115 episodes	AB 96 episodes	Cystitis 19 episodes	*P*[#]
Age (years; median [IQR])	58 (50-67)	58 (50.3-66.8)	58 (49-68)	0.94
Age >60 years	47 (40.9)	40 (41.2)	7 (36.8)	0.69
Female patients	71 (61.7)	57 (59.4)	14 (73.7)	0.24
Male patients	44 (38.3)	39 (40.6)	5 (26.3)	0.24
Diabetes mellitus	11 (9.6)	9 (9.4)	2 (10.5)	1.00
Charlson Comorbidity Index (median [IQR])	3 (3-5)	3 (3-5)	4 (3-5)	0.43
Months from transplantation (median [IQR])	14 (4-77)	15 (3.3-76.8)	11 (4-138)	0.90
< 2 months from transplantation	21 (18.3)	18 (18.8)	3 (15.8)	1.00
< 3 months from transplantation	28 (24.3)	24 (25.0)	4 (21.1)	1.00
< 6 months from transplantation	41 (35.7)	34 (35.4)	7 (36.8)	0.91
< 12 months from transplantation	56 (48.7)	45 (46.9)	11 (57.9)	0.38
Previous kidney transplantation	10 (8.7)	9 (9.4)	1 (5.3)	1.00
Living donor	11 (9.5)	10 (10.5)	1 (5.3)	0.69
Induction therapy within 3 previous months:	73 (63.5)	62 (64.6)	11 (57.9)	0.58
- Thymoglobulin	26 (22.6)	21 (21.9)	5 (26.3)	0.76
- Basiliximab	41 (35.7)	35 (36.5)	6 (31.6)	0.75
- Daclizumab	6 (5.2)	6 (6.3)	0 (0.0)	0.59
Current immunosuppression:				
- Corticosteroids	107 (93.0)	89 (92.7)	18 (94.7)	1.00
- Tacrolimus	107 (93.0)	89 (92.7)	18 (94.7)	1.00
- MMF	90 (78.3)	77 (80.2)	13 (68.4)	0.36
- mTOR inhibitors	8 (6.9)	7 (7.3)	1 (5.3)	1.00
- Cyclosporine	4 (3.4)	3 (3.1)	1 (5.3)	0.52
Acute rejection within the previous 6 months	11 (9.6)	8 (8.3)	3 (15.8)	0.39
Rejection treatment in the previous 6 months:				
- Corticosteroid's bolus	9 (7.9)	6 (6.3)	3 (15.8)	1.00
- Plasmapheresis	1 (0.9)	0 (0.0)	1 (5.3)	0.27
- Thymoglobulin	1 (0.9)	0 (0.0)	1 (5.3)	0.27
Creatinine (mg/dL) - median (IQR)	1.57 (1.21-1.57)	1.54 (1.22-1.96)	1.63 (1.14-1.95)	0.92
Bacteriuria within the previous 6 months	57 (49.6)	46 (47.9)	11 (57.9)	0.43
Antibiotic use within the previous 3 months	48 (41.7)	39 (40.6)	9 (47.3)	0.59
- Quinolones[*]	11 (9.6)	8 (8.3)	3 (15.8)	0.39
- Amoxicillin-clavulanate	6 (5.2)	5 (15.6)	1 (5.3)	1.00
- Fosfomycin	14 (12.2)	11 (11.5)	3 (15.8)	0.70
- Cephalosporins[**]	15 (13.0)	13 (13.5)	2 (10.5)	1.00
- Others[***]	6 (5.2)	5 (5.2)	1 (5.3)	0.42
Urinary pH - median (IQR)	6 (6-6.5)	6 (6-6.5)	6 (6-6.5)	0.46
Urine pH ≤6	60 (52.2)	48 (50.0)	12 (63.2)	0.29
Antibiotic therapy of the UTI episodes				
- Fosfomycin	88 (76.5)	75 (78.1)	13 (68.4)	0.38
- Ciprofloxacin	27 (23.5)	21 (21.9)	6 (31.6)	0.38

IQR: Interquartile range; MMF: Mycophenolate mofetil; mTOR inhibitors: Sirolimus, everolimus; ESBL: Extended spectrum beta-lactamases. [*]Quinolones: ciprofloxacin or levofloxacin; [**]Cephalosporins: cefixime or cefuroxime; [***]Others: Ertapenem and cloxacillin. [#]Comparison between AB and cystitis, through chi-square or Fisher exact tests.

Supplementary Table S2. Demographics and characteristics of the 115 kidney transplant recipients with urinary tract infection by *Escherichia coli* according the initial urine pH.

**Variables**	**115 episodes N (%)**	**60 episodes Urine pH ≤6 N (%)**	**55 episodes Urine pH >6 N (%)**	***P*[#]**
Age (years; median [IQR])	58 (50-67)	55.5 (47-61)	64 (54-72)	**<0.01**
Age >60 years	47 (40.9)	17 (28.3)	30 (54.5)	**0.004**
Female patients	71 (61.7)	40 (66.7)	31 (56.4)	0.26
Male patients	44 (38.3)	20 (33.3)	24 (43.6)	0.26
Diabetes mellitus	11 (9.6)	4 (6.7)	7 (12.7)	0.27
Charlson Comorbidity Index (median [IQR])	3 (3-5)	3 (2-5)	4 (3-5)	0.05
Months from transplantation (median [IQR])	14 (4-77)	17.5 (3.3-120)	11 (4-46)	0.47
< 2 months from transplantation	21 (18.3)	13 (21.7)	8 (14.5)	0.32
< 3 months from transplantation	28 (24.3)	15 (25.0)	13 (23.6)	0.87
< 6 months from transplantation	41 (38.7)	20 (33.3)	21 (38.2)	0.59
< 12 months from transplantation	56 (48.7)	26 (43.3)	30 (54.5)	0.23
Previous kidney transplantation	10 (8.7)	6 (10.0)	4 (7.3)	0.75
Living donor	11 (9.6)	7 (11.7)	4 (7.3)	0.53
Induction therapy within 3 previous months:	73 (63.5)	35 (58.3)	38 (69.1)	0.96
- Thymoglobulin	26 (22.6)	14 (23.3)	12 (21.8)	1.00
- Basiliximab	41 (35.7)	18 (30.0)	23 (41.8)	0.17
- Daclizumab	6 (5.2)	3 (5.0)	3 (5.5)	1.00
Current immunosuppression:				
- Corticosteroids	107 (93.0)	55 (91.7)	52 (94.5)	0.72
- Tacrolimus	107 (93.0)	56 (93.3)	51 (92.7)	1.00
- MMF	90 (78.3)	49 (81.7)	41 (74.5)	0.36
- mTOR inhibitors	8 (7.0)	3 (5.0)	5 (9.1)	0.48
- Cyclosporine	4 (3.5)	3 (5.0)	1 (1.8)	0.62
Acute rejection within the previous 6 months	11 (9.6)	4 (6.7)	7 (12.7)	0.27
Rejection treatment in the previous 6 months:				
- Corticosteroid's bolus	9 (7.8)	3 (5.0)	6 (10.9)	1.00
- Plasmapheresis	1 (0.9)	0 (0.0)	1 (1.8)	1.00
- Thymoglobulin	1 (0.9)	0 (0.0)	1 (1.8)	1.00
Creatinine (mg/dL; median [IQR])	1.57 (1.21-1.95)	1.56 (1.17-2.15)	1.58 (1.29-1.87)	0.83
Bacteriuria within the previous 6 months	57 (49.6)	25 (41.7)	32 (58.2)	0.08
Cystitis	19 (16.5)	12 (20.0)	7 (12.7)	0.29
Asymptomatic bacteriuria	96 (83.5)	48 (80.0)	48 (87.3)	0.29
Antibiotic therapy of the UTI episodes				
- Fosfomycin	88 (76.5)	47 (78.3)	41 (74.5)	0.63
- Ciprofloxacin	27 (23.5)	13 (21.7)	14 (25.5)	0.63

IQR: Interquartile range; MMF: Mycophenolate mofetil; mTOR inhibitors: Sirolimus, everolimus; ESBL: Extended spectrum beta-lactamases; [*]Quinolones: ciprofloxacin or levofloxacin; [**]Cephalosporins: cefixime or cefuroxime; [***]Others: Ertapenem, cloxacillin, cotrimoxazole and rifaximine. [#]Comparison between initial urine pH, through chi-square or Fisher exact tests.

Supplementary Table S3. Demographics and characteristics of the 115 kidney transplant recipients with urinary tract infection by *Escherichia coli* according symptomatic UTI during one-month follow-up.

**Variables**	**115 episodes N (%)**	**Symptomatic UTI during one-month follow-up**		***P***[#]
		**Yes (N=8)**	**No (N=107)**	
Age (years; median [IQR])	58 (50-67)	59.5 (44-64)	58 (51-68)	0.70
Age >60 years	47 (40.9)	4 (50.0)	43 (40.2)	0.71
Female patients	71 (61.7)	6 (75.0)	65 (60.7)	0.71
Male patients	44 (38.3)	2 (25.0)	42 (39.3)	0.71
Diabetes mellitus	11 (9.6)	1 (12.5)	10 (9.3)	0.57
Charlson Comorbidity Index (median [IQR])	3 (3-5)	4.5 (3.3-5.8)	3 (3-5)	0.12
Months from transplantation (median [IQR])	14 (4-77)	24 (2.5-116)	13 (4-77)	1.00
< 2 months from transplantation	21 (18.3)	2 (25.0)	19 (17.8)	0.64
< 3 months from transplantation	28 (24.3)	2 (25.0)	26 (24.3)	1.00
< 6 months from transplantation	41 (38.7)	2 (25.0)	39 (36.4)	0.71
< 12 months from transplantation	56 (48.7)	3 (37.5)	53 (49.5)	0.72
Previous kidney transplantation	10 (8.7)	1 (12.5)	9 (8.4)	0.53
Living donor	11 (9.6)	1 (12.5)	10 (9.3)	0.57
Induction therapy within 3 previous months:	73 (63.5)	4 (50.0)	69 (64.5)	0.72
- Thymoglobulin	26 (22.6)	1 (12.5)	25 (23.4)	0.68
- Basiliximab	41 (35.7)	3 (37.5)	38 (35.5)	1.00
- Daclizumab	6 (5.2)	0 (0.0)	6 (5.6)	1.00
Current immunosuppression:				
- Corticosteroids	107 (93.0)	8 (100.0)	99 (92.5)	1.00
- Tacrolimus	107 (93.0)	7 (87.5)	100 (93.5)	0.45
- MMF	90 (78.3)	6 (75.0)	84 (78.5)	1.00
- mTOR inhibitors	8 (7.0)	0 (0.0)	8 (7.5)	1.00
- Cyclosporine	4 (3.5)	0 (0.0)	4 (3.7)	1.00
Acute rejection within the previous 6 months	11 (9.6)	1 (12.5)	10 (9.3)	0.57
Rejection treatment in the previous 6 months:				
- Corticosteroid's bolus	9 (7.8)	1 (12.5)	8 (7.5)	1.00
- Plasmapheresis	1 (0.9)	0 (0.0)	1 (0.9)	1.00
- Thymoglobulin	1 (0.9)	0 (0.0)	1 (0.9)	1.00
Creatinine (mg/dL; median [IQR])	1.57 (1.21-1.95)	2.01 (1.38-2.55)	1.54 (1.21-1.89)	0.17
Cystitis	19 (16.5)	4 (50.0)	15 (14.0)	**0.025**
Asymptomatic bacteriuria	96 (83.5)	4 (50.0)	92 (86.0)	**0.025**
Urinary pH - median (IQR)	6 (6-6.5)	6 (6-6)	6.5 (6-6.5)	**0.017**
Urine pH ≤6	60 (52.2)	8 (100.0)	52 (48.6)	**0.006**
Antibiotic therapy of the UTI episodes				
- Fosfomycin	88 (76.5)	7 (87.5)	81 (75.7)	0.68
- Ciprofloxacin	27 (23.5)	1 (12.5)	26 (24.3)	0.68

IQR: Interquartile range; MMF: Mycophenolate mofetil; mTOR inhibitors: Sirolimus, everolimus; ESBL: Extended spectrum beta-lactamases. [#]Comparison between symptomatic UTI, through chi-square or Fisher exact tests.

Supplementary Table S4. Demographics and characteristics of the 69 kidney transplant recipients (KTR) with urinary tract infection (UTI) by *Klebsiella pneumoniae*, and according the asymptomatic bacteriuria (AB) and cystitis episodes.

**Variables**	***K. pneumoniae* 69 episodes**	**AB 36 episodes**	**Cystitis 33 episodes**	***P*** #
Age (years; median [IQR])	61 (50-69)	62.5 (53.3-70)	61 (47.5-68.5)	0.657
Female patients	42 (60.9)	21 (58.3)	21 (63.6)	0.652
Charlson Comorbidity Index – median (IQR)	5 (3-5)	4.5 (3.3-5.8)	5 (3-5)	0.508
Months from transplantation (median [IQR])	6 (1-77)	5 (1-50)	7 (3-99.5)	0.201
< 2 months from transplantation	21 (30.4)	14 (38.9)	7 (21.2)	0.200
Previous kidney transplantation	8 (11.6)	5 (13.9)	3 (9.1)	0.712
Living donor	6 (8.7)	3 (8.3)	3 (9.1)	1.000
Induction therapy within 3 previous months:	28 (40.6)	11 (30.6)	17 (51.4)	0.325
- Thymoglobulin	12 (17.4)	4 (11.1)	8 (24.2)	0.151
- Basiliximab	14 (20.3)	6 (16.7)	8 (24.2)	0.434
- Daclizumab	2 (2.9)	1 (2.8)	1 (3.0)	1.000
Current immunosuppression:				
- Corticosteroids	58 (84.1)	29 (80.6)	29 (87.9)	0.406
- Tacrolimus	64 (92.8)	34 (94.4)	30 (90.9)	0.665
- MMF	54 (78.3)	30 (83.3)	24 (72.7)	0.286
- mTOR inhibitors	4 (5.8)	3 (8.3)	1 (3.0)	0.615
- Cyclosporine	3 (4.3)	1 (2.8)	2 (6.1)	0.603
Acute rejection within the previous 6 months	0 (0.0)	0 (0.0)	0 (0.0)	---
Creatinine (mg/dL; median [IQR])	1.56 (1.25-1.99)	1.49 (1.24-1.97)	1.69 (1.24-2.08)	0.459
Bacteriuria within the previous 6 months	49 (71.0)	24 (66.7)	25 (75.8)	0.406
Antibiotic use within the previous 3 months	30 (43.5)	14 (38.9)	16 (48.5)	0.425
- Quinolones*	3 (4.3)	1 (2.8)	2 (6.1)	0.603
- Amoxicillin-clavulanate	8 (11.6)	5 (13.9)	3 (9.1)	0.712
- Fosfomycin	10 (14.5)	5 (13.9)	5 (15.2)	1.000
- Cephalosporins**	7 (10.1)	2 (5.6)	5 (15.2)	0.247
- Others***	2 (2.9)	1 (2.8)	1 (3.0)	0.731
Urinary pH (median [IQR])	6.5 (6-6.5)	6.5 (6-6.5)	6.5 (6-6.5)	0.979
Baseline antibiotic resistance:				
- Cotrimoxazole	42 (60.9)	24 (66.7)	18 (54.5)	0.116
- Ciprofloxacin	20 (26.1)	10 (27.8)	10 (30.3)	0.811
- Amoxicillin-clavulanate	15 (21.7)	8 (22.2)	7 (21.2)	0.973
- Fosfomycin	8 (8.7)	4 (11.1)	4 (12.1)	1.000
- Cephalosporins****	21 (30.4)	10 (27.8)	11 (33.3)	0.671
- ESBL-production	20 (29.0)	9 (25.0)	11 (33.3)	0.446
Antibiotic therapy of the UTI episodes				
- Fosfomycin	46 (66.7)	25 (69.4)	21 (63.6)	0.609
- Ciprofloxacin	23 (33.3)	11 (30.6)	12 (36.4)	0.609

IQR: Interquartile range; MMF: Mycophenolate mofetil; mTOR inhibitors: Sirolimus, everolimus; ESBL: Extended spectrum beta-lactamases; *Quinolones: ciprofloxacin or levofloxacin; **Cephalosporins: cefixime or cefuroxime; ***Others: Ertapenem and rifaximine; ****Cephalosporins: cefuroxime, cefotaxime, ceftazidime, cefixime or cefepime. #Comparison between AB and cystitis.

Supplementary Table S5. Demographics and characteristics of the 69 kidney transplant recipients with urinary tract infection by *Klebsiella pneumoniae* according the initial urine pH.

**Variables**	**69 episodes N (%)**	**29 episodes Urine pH ≤6 N (%)**	**40 episodes Urine pH >6 N (%)**	***P*[#]**
Age (years; median [IQR])	61 (50-69)	62 (55.5-69.9)	59.5 (45.5-69.75)	0.38
Age >60 years	36 (52.2)	16 (55.2)	20 (50.0)	0.67
Female patients	42 (60.9)	17 (58.6)	25 (62.5)	0.74
Male patients	27 (40.1)	12 (41.4)	15 (37.5)	0.74
Diabetes mellitus	29 (42.0)	13 (44.8)	16 (40.0)	0.69
Charlson Comorbidity Index (median [IQR])	5 (3-5)	5 (4-5)	4.5 (3-5.75)	0.46
Months from transplantation (median [IQR])	6 (1-77)	5 (0.5-44.5)	7.5 (2.25-88.25)	0.13
< 2 months from transplantation	21 (30.4)	11 (37.9)	10 (25.0)	0.25
< 3 months from transplantation	29 (42.0)	14 (48.3)	15 (37.5)	0.37
< 6 months from transplantation	35 (50.7)	17 (58.6)	18 (45.0)	0.26
< 12 months from transplantation	42 (60.9)	20 (69.0)	22 (55.0)	0.24
Previous kidney transplantation	8 (11.6)	3 (10.3)	5 (12.5)	0.78
Living donor	6 (8.7)	2 (6.9)	4 (10.0)	1.000
Induction therapy within 3 previous months:	28 (40.6)	16 (55.2)	12 (30.0)	0.22
- Thymoglobulin	12 (17.4)	7 (24.1)	5 (12.5)	0.21
- Basiliximab	14 (20.3)	8 (27.6)	6 (15.0)	0.20
- Daclizumab	2 (2.9)	1 (3.4)	1 (2.5)	1.000
Current immunosuppression:				
- Corticosteroids	58 (84.1)	27 (93.1)	31 (77.5)	0.10
- Tacrolimus	64 (92.8)	28 (96.6)	36 (90.0)	0.39
- MMF	54 (78.3)	23 (79.9)	31 (77.5)	0.86
- mTOR inhibitors	4 (5.8)	1 (3.4)	3 (7.5)	0.63
- Cyclosporine	3 (4.3)	1 (3.4)	2 (5.0)	1.000
Acute rejection within the previous 6 months	0 (0.0)	0 (0.0)	0 (0.0)	Undefined
Creatinine (mg/dL; median [IQR])	1.56 (1.25-1.99)	1.69 (1.35-2.09)	1.50 (1.22-1.88)	0.10
Bacteriuria within the previous 6 months	49 (71.0)	3 (10.3)	8 (20.0)	0.39
Cystitis	33 (47.8)	14 (48.3)	19 (47.5)	0.95
Asymptomatic bacteriuria	36 (52.2)	15 (51.7)	21 (52.5)	0.95
Antibiotic therapy of the UTI episodes				
- Fosfomycin	46 (66.7)	16 (55.2)	30 (75.0)	0.09
- Ciprofloxacin	23 (33.3)	13 (44.8)	10 (25.0)	0.09

IQR: Interquartile range; MMF: Mycophenolate mofetil; mTOR inhibitors: Sirolimus, everolimus; ESBL: Extended spectrum beta-lactamases; [*]Quinolones: ciprofloxacin or levofloxacin; [**]Cephalosporins: cefixime or cefuroxime; [***]Others: Ertapenem and rifaximine; [****]Cephalosporins: cefuroxime, cefotaxime, ceftazidime, cefixime or cefepime. [#]Comparison between initial urine pH, through chi-square or Fisher exact tests.

Supplementary Table S6. Demographics and characteristics of the 69 kidney transplant recipients with urinary tract infection by *Klebsiella pneumoniae* according symptomatic UTI during one-month follow-up.

**Variables**	**69 episodes N (%)**	**Symptomatic UTI during one-month follow-up**		$P^{\#}$
		**Yes (N=8)**	**No (N=61)**	
Age (years; median [IQR])	61 (50-69)	52 (43.3-61.5)	62 (53-70)	0.05
Age >60 years	36 (52.2)	2 (25.0)	34 (55.7)	0.14
Female patients	42 (60.9)	7 (87.5)	35 (57.4)	0.14
Male patients	27 (40.1)	1 (12.5)	26 (42.6)	0.14
Diabetes mellitus	29 (42.0)	5 (62.5)	24 (39.3)	0.27
Charlson Comorbidity Index (median [IQR])	5 (3-5)	3.5 (2-4.8)	5 (3-5.5)	**0.045**
Months from transplantation (median [IQR])	6 (1-77)	5 (1.25-134.8)	6 (1-61)	0.81
< 2 months from transplantation	21 (30.4)	3 (37.5)	18 (29.5)	0.69
< 3 months from transplantation	29 (42.0)	4 (50.0)	25 (41.0)	0.71
< 6 months from transplantation	35 (50.7)	4 (50.0)	31 (50.8)	1.00
< 12 months from transplantation	42 (60.9)	5 (62.5)	37 (60.7)	1.00
Previous kidney transplantation	8 (11.6)	1 (12.5)	7 (11.5)	1.00
Living donor	6 (8.7)	1 (12.5)	5 (8.2)	0.54
Induction therapy within 3 previous months:	28 (40.6)	3 (37.5)	25 (41.0)	0.24
- Thymoglobulin	12 (17.4)	3 (37.5)	9 (14.8)	0.14
- Basiliximab	14 (20.3)	0 (0.0)	(14 (23.0)	0.19
- Daclizumab	2 (2.9)	0 (0.0)	2 (3.3)	1.00
Current immunosuppression:				
- Corticosteroids	58 (84.1)	7 (87.5)	51 (83.6)	1.00
- Tacrolimus	64 (92.8)	7 (87.5)	57 (93.4)	0.47
- MMF	54 (78.3)	5 (62.5)	49 (80.3)	0.36
- mTOR inhibitors	4 (5.8)	1 (12.5)	3 (4.9)	0.40
- Cyclosporine	3 (4.3)	0 (0.0)	3 (4.9)	1.00
Acute rejection within the previous 6 months	0 (0.0)	0 (0.0)	0 (0.0)	Undefined
Creatinine (mg/dL; median [IQR])	1.56 (1.25-1.99)	1.14 (0.81-1.69)	1.6 (1.31-2.03)	0.06
Cystitis	33 (47.8)	6 (75.0)	27 (44.3)	0.14
Asymptomatic bacteriuria	36 (52.2)	2 (25.0)	34 (55.7)	0.14
Urinary pH - median (IQR)	6.5 (6-6.5)	6.25 (6-6.88)	6.5 (6-6.5)	0.79
Urine pH ≤6	29 (42.0)	4 (50.0)	25 (41.0)	0.71
Antibiotic therapy of the UTI episodes				
- Fosfomycin	46 (66.7)	7 (87.5)	39 (63.9)	0.25
- Ciprofloxacin	23 (33.3)	1 (12.5)	22 (36.1)	0.25

IQR: Interquartile range; MMF: Mycophenolate mofetil; mTOR inhibitors: Sirolimus, everolimus; ESBL: Extended spectrum beta-lactamases; *Quinolones: ciprofloxacin or levofloxacin; **Cephalosporins: cefixime or cefuroxime; ***Others: Ertapenem and rifaximine; ****Cephalosporins: cefuroxime, cefotaxime, ceftazidime, cefixime or cefepime. $^{\#}$Comparison between symptomatic UTI, through chi-square or Fisher exact tests.

Supplementary Table S7. Symptomatic urinary tract infection (UTI) during one-month follow-up according the acidic urine pH, after fosfomycin or ciprofloxacin therapy of UTI by *Escherichia coli* or *Klebsiella pneumoniae* (N=184).

		**Symptomatic UTI during one-month follow-up, N (%)**		***P*[#]**
		**Yes**	**No**	
**Total Cohort (N=184)**				
**Urine pH ≤6**	Total (N=89)	12/16 (75.0)	77/168 (45.8)	**0.027**
	Episodes treated with fosfomycin (N=63)	10/14 (71.4)	53/120 (44.2)	0.05
	Episodes treated with ciprofloxacin (N=26)	2/2 (100.0)	24/48 (50.0)	0.491
**Patients with age >60 years (N=81)**				
**Urine pH ≤6**	Total (N=33)	5/6 (83.3)	28/77 (36.4)	0.07
	Episodes treated with fosfomycin (N=21)	3/4 (75.0)	18/51 (35.3)	0.15
	Episodes treated with ciprofloxacin (N=12)	2/2 (100.0)	10/26 (38.5)	0.41
**Patients with age ≤60 years (N=103)**				
**Urine pH ≤6**	Total (N=56)	7/10 (70.0)	49/91 (54.3)	0.34
	Episodes treated with fosfomycin (N=42)	7/10 (70.0)	35/69 (50.7)	0.33
	Episodes treated with ciprofloxacin (N=14)	0/0 (0.0)	14/22 (63.6)	-
**Patients with initial cystitis (N=52)**				
**Urine pH ≤6**	Total (N=26)	7/10 (70.0)	19/42 (54.3)	0.29
	Episodes treated with fosfomycin (N=15)	5/8 (62.5)	10/26 (38.5)	0.42
	Episodes treated with ciprofloxacin (N=11)	2/2 (100.0)	9/16 (56.3)	0.50
**Patients with initial asymptomatic bacteriuria (N=132)**				
**Urine pH ≤6**	Total (N=63)	5/6 (83.3)	58/126 (46.0)	0.10
	Episodes treated with fosfomycin (N=48)	5/6 (83.3)	43/94 (45.7)	0.10
	Episodes treated with ciprofloxacin (N=15)	0/0 (0.0)	15/32 (46.9)	-

[#] Chi-square or Fisher exact tests.

Supplementary Table S8. Symptomatic urinary tract infection (UTI) during one-month follow-up according the acidic urine pH, after fosfomycin or ciprofloxacin therapy of UTI by *Escherichia coli*.

		Symptomatic UTI during one-month follow-up, N (%)		$P^{\#}$
		**Yes**	**No**	
**Total Cohort (N=115)**				
**Urine pH ≤6**	Total (N=60)	8/8 (100.0)	52/107 (48.6)	**0.006**
	Episodes treated with fosfomycin (N=47)	7/7 (100.0)	40/81 (49.4)	**0.013**
	Episodes treated with ciprofloxacin (N=13)	1/1 (100.0)	12/26 (46.2)	0.481
**Patients with age >60 years (N=47)**				
**Urine pH ≤6**	Total (N=17)	4/4 (100.0)	13/43 (30.2)	**0.013**
	Episodes treated with fosfomycin (N=14)	3/3 (100.0)	11/31 (35.5)	**0.061**
	Episodes treated with ciprofloxacin (N=3)	1/1 (100.0)	2/12 (16.7)	0.231
**Patients with age ≤60 years (N=68)**				
**Urine pH ≤6**	Total (N=43)	4/4 (100.0)	39/64 (60.9)	0.289
	Episodes treated with fosfomycin (N=33)	4/4 (100.0)	29/50 (58.0)	0.148
	Episodes treated with ciprofloxacin (N=10)	0/0 (0.0)	10/14 (71.4)	-
**Patients with initial cystitis (N=19)**				
**Urine pH ≤6**	Total (N=12)	4/4 (100.0)	8/15 (53.3)	0.245
	Episodes treated with fosfomycin (N=9)	3/3 (100.0)	6/10 (60.0)	0.497
	Episodes treated with ciprofloxacin (N=3)	1/1 (100.0)	2/5 (40.0)	1.000
**Patients with initial asymptomatic bacteriuria (N=96)**				
**Urine pH ≤6**	Total (N=48)	4/4 (100.0)	44/92 (47.8)	0.117
	Episodes treated with fosfomycin (N=38)	4/4 (100.0)	34/71 (47.9)	0.115
	Episodes treated with ciprofloxacin (N=10)	0/0 (0.0)	10/21 (47.6)	-

[#] Chi-square or Fisher exact tests.

Supplementary Table S9. Symptomatic urinary tract infection (UTI) during one-month follow-up according the acidic urine pH, after fosfomycin or ciprofloxacin therapy of UTI by *Klebsiella pneumoniae* (N=69).

		**Symptomatic UTI during one-month follow-up, N (%)**		***P*[#]**
		**Yes**	**No**	
**Total Cohort (N=69)**				
**Urine pH ≤6**	Total (N=29)	4/8 (50.0)	25/61 (41.0)	0.71
	Episodes treated with fosfomycin (N=16)	3/7 (42.9)	13/39 (33.3)	0.68
	Episodes treated with ciprofloxacin (N=13)	1/1 (100.0)	12/22 (54.5)	1.00
**Patients with age >60 years (N=36)**				
**Urine pH ≤6**	Total (N=16)	1/2 (50.0)	15/34 (44.1)	1.00
	Episodes treated with fosfomycin (N=7)	0/1 (0.0)	7/20 (35.0)	1.00
	Episodes treated with ciprofloxacin (N=9)	1/1 (0.0)	8/14 (57.1)	1.00
**Patients with age ≤60 years (N=33)**				
**Urine pH ≤6**	Total (N=13)	3/6 (50.0)	10/27 (37.3)	0.66
	Episodes treated with fosfomycin (N=9)	3/6 (50.0)	6/19 (31.6)	0.63
	Episodes treated with ciprofloxacin (N=4)	0/0 (100.0)	4/8 (50.0)	-
**Patients with initial cystitis (N=33)**				
**Urine pH ≤6**	Total (N=14)	3/6 (50.0)	11/27 (40.1)	1.00
	Episodes treated with fosfomycin (N=6)	2/5 (40.0)	4/16 (25.0)	0.56
	Episodes treated with ciprofloxacin (N=8)	1/1 (100.0)	7/11 (63.6)	1.00
**Patients with initial asymptomatic bacteriuria (N=36)**				
**Urine pH ≤6**	Total (N=15)	1/2 (50.0)	14/34 (41.2)	1.00
	Episodes treated with fosfomycin (N=10)	1/2 (50.0)	9/23 (39.1)	1.00
	Episodes treated with ciprofloxacin (N=5)	0/0 (0.0)	5/11 (45.5)	-

[#] Chi-square or Fisher exact tests.

Supplementary Table S10. Microbiological and clinical outcomes, in patients with acidic (pH ≤6) *vs*. non-acidic (pH >6) urine, after fosfomycin or ciprofloxacin therapy of urinary tract infection (UTI) regardless of aetiology.

**Variable**			**Urinary pH ≤6 N (%)**	**Urinary pH >6 N (%)**	***P*[#]**
**UTI episodes (N=184)**					
Microbiological cure during one-month follow-up	Total		47/89 (52.8)	53/95 (55.8)	0.706
	-	Treated with fosfomycin	33/63 (52.4)	35/71 (57.4)	0.848
	-	Treated with ciprofloxacin	14/26 (53.9)	18/24 (75.0)	0.287
	Cystitis at basal episode		9/26 (34.6)	15/26 (57.7)	0.263
	-	Treated with fosfomycin	4/15 (26.7)	9/19 (47.4)	0.339
	-	Treated with ciprofloxacin	5/11 (45.5)	6/7 (85.7)	0.373
	Asymptomatic bacteriuria at basal episode		38/63 (60.3)	38/69 (55.1)	0.839
	-	Treated with fosfomycin	29/48 (60.4)	26/52 (50.0)	0.529
	-	Treated with ciprofloxacin	9/15 (60.0)	12/17 (70.6)	0.512
Symptomatic UTI during one-month follow-up	**Total**		**12/89 (13.5)**	**4/95 (4.2)**	**0.052**
	-	Treated with fosfomycin	10/63 (15.9)	4/71 (5.6)	0.138
	-	Treated with ciprofloxacin	2/26 (7.7)	0/24 (0.0)	0.387
	Cystitis at basal episode		7/26 (26.9)	3/26 (11.5)	0.220
	-	Treated with fosfomycin	5/15 (33.3)	3/19 (15.8)	0.306
	-	Treated with ciprofloxacin	2/11 (18.2)	0/7 (0.0)	0.527
	Asymptomatic bacteriuria at basal episode		5/63 (7.9)	1/69 (1.5)	0.145
	-	Treated with fosfomycin	5/48 (10.4)	1/52 (1.9)	0.234
	-	Treated with ciprofloxacin	0/15 (0.0)	0/17 (0.0)	0.600
Symptomatic UTI during six-month follow-up	Total		14/89 (15.7)	15/95 (15.8)	0.825
	-	Treated with fosfomycin	11/63 (17.5)	13/71 (18.3)	0.921
	-	Treated with ciprofloxacin	3/26 (11.5)	2/24 (8.3)	0.299
	Cystitis at basal episode		3/26 (11.5)	5/26 (19.2)	0.925
	-	Treated with fosfomycin	1/15 (6.6)	3/19 (15.8)	0.871
	-	Treated with ciprofloxacin	2/11 (18.2)	2/7 (28.6)	0.730
	Asymptomatic bacteriuria at basal episode		11/63 (17.5)	10/69 (14.5)	0.730
	-	Treated with fosfomycin	10/48 (20.8)	10/52 (19.2)	0.884
	-	Treated with ciprofloxacin	1/15 (0.0)	0/17 (0.0)	0.600

[#] Chi-square or Fisher tests

Supplementary Table S11. Initial clinical, urine pH, and microbiological characteristics of patients with symptomatic *Escherichia coli* and *Klebsiella pneumoniae* UTI episodes during one-month follow-up after fosfomycin or ciprofloxacin therapy.

ID	Initial			Ciprofloxacin MIC		Fosfomycin MIC		Therapy	One-month follow-up			Ciprofloxacin MIC		Fosfomycin MIC	
	Isolate	UTI	Urine pH *	At pH 7	At pH 5	At pH 7	At pH 5		Isolate	UTI	Urine pH *	At pH 7	At pH 5	At pH 7	At pH 5
1	*E. coli*	Cystitis	6	<0.01	0.12	0.50	1	FOS	*E. coli*	Persistence/Cystitis	6	<0.01	0.06	1	0.50
2	*E. coli*	AB	6	<0.01	<0.01	0.50	2	FOS	*E. coli*	Recurrence/Cystitis	6.5	-	-	-	-
3	*E. coli*	Cystitis	6	8	>8	512	512	FOS	*E. coli*	Persistence/Cystitis	6	>8	>8	1024	512
4	*E. coli*	AB	6	8	>8	1	0.50	FOS	*E. coli*	Recurrence/APN	6	>8	>8	128	64
5	*E. coli*	Cystitis	6	0.25	2	1	1	FOS	*E. coli*	Persistence/Cystitis	6	8	>8	1	0.50
6	*E. coli*	Cystitis	6	<0.01	0.12	0.25	1	CIP	*E. coli* **	Reinfection/Cystitis	6	-	-	-	-
7	*E. coli*	AB	6	<0.01	0.12	<0.12	0.50	FOS	*E. coli*	Persistence/Cystitis	6.5	-	-	-	-
8	*E. coli* **	AB	6	<0.01	0.12	<0.12	0.50	FOS	*E. coli*	Recurrence/Cystitis	6	<0.01	0.12	2	1
1	*K. pneumoniae*	Cystitis	6	<0.01	0.25	128	64	FOS	*E. coli*	Reinfection/Cystitis	6	-	-	-	-
2	*K. pneumoniae* **	Cystitis	6	0.12	2	32	64	CIP	*K. pneumoniae* **	Recurrence/Cystitis	5.5	0.12	8	32	32
3	*K. pneumoniae*	Cystitis	6	<0.01	0.50	8	32	FOS	*E. coli* and *K. pneumoniae*	Persistence/Cystitis	6.5	-	-	-	-
4	*K. pneumoniae*	AB	5.5	<0.01	0.50	64	32	FOS	*K. pneumoniae*	Recurrence/Cystitis	5.5	0.25	0.25	32	64
5	*K. pneumoniae*	AB	6.5	0.25	4	32	32	FOS	*K. pneumoniae*	Persistence/APN	6.5	0.12	8	16	4
6	*K. pneumoniae*	Cystitis	8	<0.01	0.50	64	64	FOS	*E. coli*	Reinfection/Cystitis	7	-	-	-	-
7	*K. pneumoniae* **	Cystitis	7	>8	>8	1024	256	FOS	ND	Cystitis	7	-	-	-	-
8	*K. pneumoniae* **	Cystitis	6.5	1	>8	64	16	FOS	*K. pneumoniae* **	Recurrence/Cystitis	6	2	>8	8	32

ID: Patient identification; UTI: Urinary Tract Infection; * Acidic pH ≤6, alkaline pH >7; MIC: Minimum Inhibitory Concentration; AB: Asymptomatic bacteriuria; APN: Acute pyelonephritis; FOS: Fosfomycin; CIP: Ciprofloxacin; ** Extended spectrum beta-lactamase production; Persistence: Same bacterial isolate with no negative results in urine cultures between basal one-month follow-up; Reinfection: Different bacterial isolate in urine cultures between basal one-month follow-up; Recurrence: Same bacterial isolate with negative results in urine cultures between basal one-month follow-up; -: Isolate not available to perform MIC assays; ND: not determined (colonization).

Supplementary Table S12. Ciprofloxacin and fosfomycin MIC distribution and frequencies of low-level quinolone and fosfomycin resistance rates in the 115 isolates of *Escherichia coli* and according the AB and cystitis episodes.

	E. coli **115 episodes N (%)**	**AB 96 episodes N (%)**	**Cystitis 19 episodes N (%)**	***P*** [#]
**MIC Ciprofloxacin**				
- ≤0.01	51 (44.3)	42 (43.8)	9 (47.4)	0.807
- 0.01	12 (10.4)	8 (8.3)	4 (21.1)	0.077
- 0.03	1 (0.9)	1 (1.0)	0 (0.0)	1.000
- 0.06	9 (7.8)	8 (8.3)	1 (5.3)	0.352
- 0.12	7 (6.1)	6 (6.3)	1 (5.3)	1.000
- 0.25	2 (1.7)	1 (1.0)	1 (5.3)	0.275
- 0.50	1 (0.9)	1 (1.0)	0 (0.0)	1.000
- 1	1 (0.9)	0 (0.0)	1 (5.3)	0.148
- 2	2 (1.7)	2 (2.1)	0 (0.0)	1.000
- 4	9 (7.8)	8 (8.3)	1 (5.3)	1.000
- 8	20 (17.4)	19 (19.8)	1 (5.3)	0.299
**Resistance to ciprofloxacin**	32 (27.8)	29 (30.2)	3 (15.8)	0.391
**LLQR**	10 (8.7)	8 (8.3)	2 (10.5)	0.655
**MIC_{50}** (mg/L)	0.01	0.03	<0.01	---
**MIC_{90}** (mg/L)	8	>8	2	---
**MIC fosfomycin**				
- ≤0.12	13 (11.3)	11 (11.5)	2 (10.5)	1.000
- 0.25	25 (21.7)	22 (22.9)	3 (15.8)	1.000
- 0.50	40 (34.8)	34 (35.4)	6 (31.6)	0.614
- 1	18 (15.7)	15 (15.6)	3 (15.8)	0.729
- 2	7 (6.1)	4 (4.2)	3 (15.8)	0.276
- 8	3 (2.6)	3 (3.1)	0 (0.0)	1.000
- 16	2 (1.7)	2 (2.1)	0 (0.0)	1.000
- 32	2 (1.7)	1 (1.0)	1 (5.3)	0.275
- 128	3 (2.6)	3 (3.1)	0 (0.0)	1.000
- 512	2 (1.7)	1 (1.0)	1 (5.3)	0.275
**Resistance to fosfomycin**	9 (7.8)	7 (7.3)	2 (10.5)	0.620
**LLFR**	3 (2.6)	3 (3.1)	0 (0.0)	1.000
**MIC_{50}** (mg/L)	0.50	0.50	0.50	---
**MIC_{90}** (mg/L)	8	2	2	---

AB: Asymptomatic Bacteriuria; MIC: Minimum Inhibitory Concentration; Resistance to ciprofloxacin: MIC > 0.5 mg/L; LLQR: Low-Level Quinolone Resistance (0.06<MIC≤0.50); MIC_{50}: MIC of 50% of isolates; MIC_{90}: MIC of 90% of isolates; Resistance to fosfomycin: MIC>8 mg/L; LLFR: Low-Level Fosfomycin Resistance (4<MIC≤8); [#]Comparison between AB and cystitis.

Supplementary Table S13. Ciprofloxacin and fosfomycin MIC distribution and frequencies of low-level quinolone and fosfomycin resistance rates in the 69 isolates of *Klebsiella pneumoniae* and according the AB and cystitis episodes.

	K. pneumoniae **69 episodes N (%)**	**AB 36 episodes N (%)**	**Cystitis 33 episodes N (%)**	***P*** [#]
**MIC Ciprofloxacin**				
- ≤0.01	24 (34.8)	10 (27.8)	14 (42.4)	0.356
- 0.01	1 (1.5)	0 (0.0)	1 (3.1)	1.000
- 0.06	3 (4.3)	1 (2.7)	2 (6.0)	1.000
- 0.12	5 (7.2)	4 (11.1)	1 (3.1)	0.198
- 0.25	8 (11.6)	4 (11.1)	4 (12.1)	1.000
- 0.50	5 (7.2)	4 (11.1)	1 (3.1)	0.198
- 1	9 (13.0)	6 (16.7)	3 (9.1)	0.481
- 2	4 (5.8)	2 (5.6)	2 (6.0)	0.614
- 4	4 (5.8)	2 (5.6)	2 (6.0)	1.000
- 8	1 (1.5)	1 (2.7)	0.0 (0)	0.493
- >8	5 (7.2)	2 (5.6)	3 (9.1)	1.000
**Resistance to ciprofloxacin**	23 (33.3)	13 (36.1)	10 (30.3)	0.865
**LLQR**	13 (18.8)	8 (22.2)	5 (15.2)	0.326
**MIC_{50}** (mg/L)	0.25	0.25	0.06	---
**MIC_{90}** (mg/L)	4	4	4	---
**MIC fosfomycin**				
- 4	3 (4.3)	2 (5.6)	1 (3.1)	0.614
- 8	11 (16.0)	3 (8.3)	8 (24.2)	0.111
- 16	15 (21.7)	7 (19.4)	8 (24.2)	0.163
- 32	20 (29.0)	13 (36.1)	7 (21.2)	0.095
- 64	9 (13.0)	5 (13.9)	4 (12.1)	0.734
- 128	2 (2.9)	1 (2.7)	1 (3.1)	1.000
- 256	2 (2.9)	2 (5.6)	0.0 (0)	0.239
- 512	1 (1.5)	1 (2.7)	0.0 (0)	0.493
- 1024	1 (1.5)	0 (0.0)	1 (3.1)	1.000
- >1024	5 (7.2)	2 (5.6)	3 (9.1)	1.000
**Resistance to fosfomycin**	55 (79.7)	31 (86.1)	24 (72.6)	1.000
**LLFR**	NA	NA	NA	---
**MIC_{50}** (mg/L)	32	32	16	---
**MIC_{90}** (mg/L)	512	256	128	---

AB: Asymptomatic Bacteriuria; MIC: Minimum Inhibitory Concentration; Resistance to ciprofloxacin: MIC>0.5 mg/L; LLQR: Low-Level Quinolone Resistance (0.12<MIC≤0.50); MIC_{50}: MIC of 50% of isolates; MIC_{90}: MIC of 90% of isolates; Resistance to fosfomycin: MIC>8 mg/L; NA: Not applicable; [#]Comparison between AB and cystitis.

Supplementary Table S14. *In vitro* MIC distribution of ciprofloxacin at pH 5.0 (acidic) and 8.0 (alkaline) of *E. coli* and *K. pneumoniae* clinical isolates with low-level ciprofloxacin resistance at pH 7.0 (neutral).

Microorganism	Isolates (n)	pH of test	No. of isolates with the following MIC (mg/L):												MIC (mg/L)	
			<0.01	0.01	0.03	0.06	0.12	0.25	0.5	1	2	4	8	>8	50%	90%
Escherichia coli	10	5	-	-	-	-	-	-	2	-	5	-	1	2	2	≥8
		7	-	-	-	-	7	2	1	-	-	-	-	-	0.12	0.25
		8	-	-	-	2	3	1	3	1	-	-	-	-	0.12	0.5
Klebsiella pneumoniae	13	5	-	-	-	-	-	4	-	-	4	4	1	-	2	4
		7	-	-	-	-	-	8	5	-	-	-	-	-	0.25	0.5
		8	3	-	1	-	3	5	1	-	-	-	-	-	0.12	0.25

-: no isolate; S: susceptible; LLQR: Low-level quinolone resistance; R: resistant; EUCAST. European Committee on Antimicrobial Susceptibility Testing interpretative criteria (resistant. MIC > 0.5 g/L).

Supplementary Table S15. *In vitro* MIC distribution of fosfomycin at pH 5.0 (acidic) and 8.0 (alkaline) of *E. coli* clinical isolates with low-level fosfomycin resistance at pH 7.0 (neutral).

Microorganism	Isolates (n)	pH of test	No. of isolates with the following MIC (mg/L):						MIC (mg/L)	
			4	8	16	32	64	128	50%	90%
Escherichia coli	3	5	1	1	-	1	-	-	8	32
		7	-	3	-	-	-	-	8	8
		8	-	1	-	1	-	1	32	128

-: no isolate; S: susceptible; LLFR: Low-level fosfomycin resistance; R: resistant; EUCAST. European Committee on Antimicrobial Susceptibility Testing interpretative criteria (resistant. MIC > 8 g/L).

Supplementary Table S16. pH effect on the bactericidal activity of ciprofloxacin MIC, in MHB and urine, against uropathogenic *Escherichia coli* strains.

**pH**	**Time (h)**	***E. coli* Nu14**		***E. coli* HUVR 94**		***E. coli* Nu79 *gyrA* D87G**		***E. coli* Nu14 *glpT* missense mutation**	
		**MHB**	**Urine**	**MHB**	**Urine**	**MHB**	**Urine**	**MHB**	**Urine**
Neutral	2	-2.40	-2.71	-1.46	-1.20	-1.24	-1.69	-1.25	-1.65
	4	-2.65	-2.98	-2.68	-1.79	-2.28	-2.03	-1.50	-2.12
	6	-2.87	-3.30	-3.11	-2.30	-2.97	-2.79	-1.84	-2.25
	24	-5.73	-5.70	-5.15	-4.91	-5.38	-4.34	-1.08	-4.37
Acidic	2	0.33	0.18	-0.21	-0.84	-0.83	-0.40	0.16	-0.32
	4	0.96	1.42	-0.34	-1.42	-0.41	0.39	1.13	0.64
	6	2.20	2.09	2.21	2.06	2.19	0.33	1.31	1.91
	24	2.70	2.39	1.86	-4.79	3.84	1.59	3.57	1.86
Alkaline	2	-2.49	-2.20	-2.23	-1.57	-1.53	-1.51	-2.05	-1.58
	4	-2.39	-2.65	-2.85	-1.92	-2.54	-2.60	-2.27	-1.85
	6	-2.86	-2.65	-3.31	-2.27	-2.95	-2.24	-2.66	-2.31
	24	-4.44	-5.72	-5.17	-4.09	-5.41	-5.20	-4.68	-4.71

Results are represented as differences (log_{10} CFU/mL) relative to the initial time point (0 h). Green indicates a >3 log_{10} CFU/mL decrease, orange a 3 to 0 log_{10} CFU/mL decrease, and white no bacterial reduction. Ciprofloxacin MIC concentrations (mg/L): 0.03, 0.03, 0.12 and 0.01 for *E. coli* Nu14, *E. coli* HUVR 94, *E. coli* Nu79 *gyrA* D87G, and *E. coli* Nu14 *glpT* missense mutation, respectively.

Supplementary Table S17. pH effect on the bactericidal activity of fosfomycin MIC; in MHB and urine, against uropathogenic *Escherichia coli* strains.

pH	Time (h)	*E. coli* Nu14		*E. coli* HUVR 94		*E. coli* Nu79 *gyrA* D87G		*E. coli* Nu14 *glpT* missense mutation	
		MHB	Urine	MHB	Urine	MHB	Urine	MHB	Urine
Neutral	2	-3.34	-2.72	-1.27	-0.07	-2.48	-0.82	-2.24	-0.81
	4	-3.32	-3.31	-2.33	0.01	-3.75	-2.71	-2.81	-2.46
	6	-3.88	-2.34	2.94	0.00	-3.84	-1.41	-3.09	-2.41
	24	2.09	0.85	1.87	2.69	1.26	2.42	3.33	1.93
Acidic	2	-0.70	-0.19	-1.22	-1.21	-1.44	-0.34	-1.84	-2.31
	4	-2.79	-1.55	-2.12	-1.58	-1.16	0.58	-2.79	-2.78
	6	-2.27	-1.23	-1.54	-2.12	-1.66	0.16	-3.56	-2.61
	24	2.52	1.43	1.97	2.14	1.42	3.06	2.77	1,13
Alkaline	2	-2.64	-1.28	-1.43	-0.32	-1.86	-0.43	-2.31	-1.34
	4	-3.02	-0.91	-2.17	0.72	-3.96	0.45	-3.21	-1.58
	6	-2.50	-0.27	2.88	1.98	-3.99	0.96	-3.45	-1.82
	24	1.54	0.97	1.80	2.51	-0.10	2.04	3.18	1.61

Results are represented as differences (log_{10} CFU/mL) relative to the initial time point (0 h). Green indicates a >3 log_{10} CFU/mL decrease, orange a 3 to 0 log_{10} CFU/mL. Fosfomycin MIC concentrations (mg/L): 2, 0.50, 0.50 and 32 for *E. coli* Nu14, *E. coli* HUVR 94, *E. coli* Nu79 *gyrA* D87G, and *E. coli* Nu14 *glpT* missense mutation, respectively.

Supplementary Table S18. pH effect on the bactericidal activity of ciprofloxacin MIC, in MHB and urine, against uropathogenic *Klebsiella pneumoniae* strains.

pH	Time (h)	*K. pneumoniae* HUVR42		*K. pneumoniae* HUVR5		*K. pneumoniae* HUVR110		*K. pneumoniae* HUVR91	
		MHB	Urine	MHB	Urine	MHB	Urine	MHB	Urine
Neutral	2	-0.19	-0.29	-3.07	-1.49	-1.65	-1.51	-0.08	-0.38
	4	-0.18	0.19	-3.87	-2.09	-2.08	-2.38	0.01	0.41
	6	0.45	1.65	-4.08	-2.70	-2.81	-3.07	2.15	-1.83
	24	3.54	3.63	-5.47	-4.21	-3.93	-5.40	3.63	3.06
Acidic	2	-0.40	-0.17	-4.95	-4.21	-1.72	-1.64	0.53	-0.01
	4	0.87	0.40	-4.45	-4.15	-2.84	-2.60	2.55	-0.37
	6	0.28	2.18	-5.45	-5.45	-3.33	-3.30	3.40	1.62
	24	4.22	3.33	-4.95	-5.45	-4.87	-4.88	4.04	2.88
Alkaline	2	-0.14	-0.17	-1.21	-1.38	-1.58	-1.34	-0.82	-0.18
	4	-0.82	-0.03	-1.87	-1.97	-2.10	-2.16	-1.64	-0.91
	6	-1.05	1.54	-2.57	-2.71	-2.74	-2.95	-2.12	-1.10
	24	4.18	2.45	-4.77	-4.63	-4.13	-3.70	4.02	2.61

Results are represented as differences (log_{10} CFU/mL) relative to the initial time point (0 h). Green indicates a >3 log_{10} CFU/mL decrease, red a 3 to 0 log_{10} CFU/mL decrease, and white no bacterial reduction. Ciprofloxacin MIC concentrations (mg/L): 0.007, 8, 8 and 0.006 for *K. pneumoniae* HUVR42, *K. pneumoniae* HUVR5, *K. pneumoniae* HUVR110, and *K. pneumoniae* HUVR91, respectively.

Supplementary Table S19. pH effect on the bactericidal activity of fosfomycin MIC; in MHB and urine, against uropathogenic *Klebsiella pneumoniae* strains.

pH	Time (h)	*K. pneumoniae* HUVR42		*K. pneumoniae* HUVR5		*K. pneumoniae* HUVR110		*K. pneumoniae* HUVR91	
		**MHB**	**Urine**	**MHB**	**Urine**	**MHB**	**Urine**	**MHB**	**Urine**
Neutral	2	-0.72	-0.19	-2.49	-0.99	-1.58	-1.05	-0.86	-2.35
	4	-0.99	-0.90	2.80	-2.14	-1.57	-2.05	-2.53	-2.74
	6	-1.84	-1.06	-1.29	-1.55	-2.11	-2.43	-1.67	-2.08
	24	3.82	3.14	3.61	2.72	-3.66	-4.76	3.69	3.34
Acidic	2	-0.72	-0.37	-2.40	-2.41	-0.42	-0.40	-2.76	-3.51
	4	-0.82	-1.06	-2.67	-2.79	-1.37	-1.60	-2.80	-2.77
	6	-1.17	-1.13	-3.21	-2.80	-1.82	-2.44	-3.17	-2.89
	24	1.55	2.53	-3.32	-3.30	2.16	-4.33	3.94	3.31
Alkaline	2	-0.59	-0.27	-2.41	-1.76	-0.10	-0.47	-0.11	-1.04
	4	-1.14	-0.61	-2.79	-2.51	-1.56	-1.09	-1.84	-1.79
	6	-1.23	-1.01	-2.80	-0.47	-1.63	-2.69	0.57	-0.62
	24	3.69	1.93	-3.30	2.93	2.20	2.52	3.81	3.92

Results are represented as differences ($\log_{10}$ CFU/mL) relative to the initial time point (0 h). Green indicates a >3 $\log_{10}$ CFU/mL decrease, orange a 3 to 0 $\log_{10}$ CFU/mL. Fosfomycin MIC concentrations (mg/L): 4, 128, 4, and 64 for *K. pneumoniae* HUVR42, *K. pneumoniae* HUVR5, *K. pneumoniae* HUVR110, and *K. pneumoniae* HUVR91, respectively.
